# Journalism and the Hospitals

**Published:** 1914-10-10

**Authors:** 


### The Hospital

The Workers' Newspaper of Administrative Medicine and Institutional Life, Administration, National Insurance and Health.

No. 1477, Vol. LVII. SATURDAY, OCTOBER 10, 1914. PRICE ONE PENNY.

### JOURNALISM AND THE HOSPITALS.

The life of a hospital, like the life of an army—just now we can hardly escape the comparison—is a much more complex and more intricate process than the superficial observer is apt to imagine. In each case, part is on the surface and readily catches the eye. But in each case, also, much is hidden, and therefore escapes observation. For the most part, only those who have penetrated behind the scenes know how extensive is this concealed machinery. The soldiers and the guns are there for everyone to see. In the popular sense of the term they *are* the army. What is but faintly realised is that the life and activities thus expressed are the outcome of varied and complex organisations, devised and controlled by highly cultivated intelligence, and made effective by daily routine and unrecognised effort. Similarly, to many, the hospital is a building which accommodates a certain number of patients, and provides for these the services of an appropriate contingent of doctors and nurses. That underneath this result, and making it possible, there is an elaborate, unceasing, and complicated series of activities, is recognised only by the few. The patients and the doctors and the nurses, like the soldiers and the guns, seem to embody and announce a self-contained life and energy, and the directing brains and methodical and routine tasks which make this life and energy possible, are to a large extent out of the picture. In this journal we stand for the proposition that the voluntary hospital service of this country is a great national interest—an interest of vital importance to the sick and suffering, and hardly less vital as an opportunity and an education for those who may render personal service to their less fortunate brethren. Hence in these columns we try to present the whole chapter of hospital activities and hospital work, giving to each its due recognition and encouragement, and offering to our readers a full and exhaustive account of the manifold activities of hospital life. Whatever pertains to this life, however humble and obscure, cannot possibly be alien from the mind and sympathies of The Hospital.

One of the features of the work which we here cultivate is an endeavour to attract and to educate the sympathies of those whose interest in hospitals may be described as non-professional. It is impossible to overvalue the services which are daily rendered to our voluntary hospital system by men of business and affairs who willingly give time and experience to the tasks of administration. These provide, indeed, both the judgment and the guarantee without which the system would promptly come to a compendious end. We are also convinced that if the best of our laymen are to be actively interested in the management of our hospitals, they must have full information and full opportunity of appreciating what is involved in the various questions presented for their consideration.

To meet at stated intervals in a comfortable board room for the purpose of giving formal assent to propositions which have been prepared in advance by duly appointed officials will never satisfy men of independence and insight. Yet these latter are the men who are essential if the business of our hospitals is to be conducted in harmony with the claims of sound principle and healthy and vigorous enterprise. To obtain the best service we must recruit the best minds, and these will only serve where they can act with judgment and independence founded on personal knowledge. It is one of our aims to supply them with this knowledge, and in this fashion to gain for our voluntary hospitals the accumulated experience and trained discretion which are essential if benevolence and goodwill are to be linked to efficient administration and organised success. In the hospital scheme of things, therefore, and as a means of promoting intelligent co-operation and interest, journalism, we may fairly claim, has its invaluable place.

Turning next to the intra-mural life of the hospitals we are promptly met by the demands associated with the existence of numerous bodies of technical and expert workers. One of these demands is certainly the possession of an opportunity for mutual communication and for the dissemination of information and ideas. A journal devoted to these ends is found, therefore, to be a necessity of the position. Without it the individual worker remains separate and detached from his fellows; he lacks the stimulus which comes from the contact of mind with mind; and he may remain ignorant of help which could readily be had for the asking. In this respect it has to be remembered that nearly all forms of work associated with hospital life take on a specialised quality. Architecture, internal arrangement, engineering, sanitary services, organisation of supplies, financial management, and domestic economy assume, in connection with hospitals, individual and characteristic features, which demand for their adequate treatment the confidence arising from actual trial and experience. It is of the first importance that all workers in each of these several departments shall be kept in touch with one another, both for mutual help and in order, also, that improvements and advances of local origin may reach and benefit the whole body. These ends we here endeavour to serve. So far as lies in our power we make it our duty to cultivate an outlook both wide in extent and practical in detail, and our columns are always open to anyone who can contribute to the general interest either information or suggestion or criticism. The common ambition of all workers is to render more and more complete the efficiency of the hospital services in our own country, and indeed in all countries, for happily the mission knows no limitation of race or nationality. In such work the journalist has this satisfaction, that he provides the opportunity for each to learn from the experience of his colleagues, while each, also, may add his thought to touch and benefit the whole.

There remain the doctors and nurses, for whose professional activities all other hospital activities exist. Without them we might have fine buildings and complete organisation, but all to no end. It is perhaps not altogether unnatural that those who have so prominent a place in the sun should at times be inclined to forget others whose work is mainly underground. Hence we do not hesitate to put in a plea for mutual recognition and respect among all hospital workers. There may be different ranks and orders, but there is only one end. We try to keep this in view, and while we strive to do something for the difficulties of each group we endeavour also to foster the mutual understanding which is essential to cordial co-operation. From many proofs we know that both doctors and nurses view our journalistic work with every sympathy, and it is our privilege often to serve them. The present great position of our hospitals is due to the happy and efficient combination of both professional and lay workers. In some modest fashion we hope to have promoted this combination, by the spread of information, by the removal of misunderstandings, and by the promotion of opportunities for mutual conference and counsel. This work we shall strive to continue, and we appeal with confidence for the support of all who recognise the national and individual importance of our existing hospital system.

